# Lauric Acid Overcomes Hypoxia-Induced Gemcitabine Chemoresistance in Pancreatic Ductal Adenocarcinoma

**DOI:** 10.3390/ijms24087506

**Published:** 2023-04-19

**Authors:** Tadataka Takagi, Rina Fujiwara-Tani, Shiori Mori, Shingo Kishi, Yukiko Nishiguchi, Takamitsu Sasaki, Ruiko Ogata, Ayaka Ikemoto, Rika Sasaki, Hitoshi Ohmori, Yi Luo, Ujjal Kumar Bhawal, Masayuki Sho, Hiroki Kuniyasu

**Affiliations:** 1 Department of Molecular Pathology, Nara Medical University, Kashihara 634-8521, Japan; 2 Department of Surgery, Nara Medical University, Kashihara 634-8522, Japan; 3 Jiangsu Province Key Laboratory of Neuroregeneration, Nantong University, 19 Qixiu Road, Nantong 226001, China; 4 Department of Biochemistry and Molecular Biology, Nihon University School of Dentistry at Matsudo, Matsudo 271-8587, Japan; 5 Department of Pharmacology, Saveetha Dental College, Saveetha Institute of Medical and Technical Sciences, Chennai 600077, India

**Keywords:** gemcitabine, drug resistance, hypoxia, mitochondria, pancreatic cancer

## Abstract

Although gemcitabine (GEM) is widely used in chemotherapy for pancreatic ductal adenocarcinoma (PDA), drug resistance restricts its clinical effectiveness. To examine the mechanism of GEM resistance, we established two GEM-resistant cell lines from human PDA cells by continuous treatment with GEM and CoCl_2_-induced chemical hypoxia. One resistant cell line possessed reduced energy production and decreased mitochondrial reactive oxygen species levels, while the other resistant cell line possessed increased stemness. In both cell lines, ethidium bromide-stained mitochondrial DNA levels decreased, suggesting mitochondrial DNA damage. Inhibition of hypoxia-inducible factor-1α in both cell lines did not restore the GEM sensitivity. In contrast, treatment of both cell types with lauric acid (LAA), a medium-chain fatty acid, restored GEM sensitivity. These results suggest that decreased energy production, decreased mitochondrial reactive oxygen species levels, and increased stemness associated with mitochondrial damage caused by GEM lead to GEM resistance, and that hypoxia may promote this process. Furthermore, forced activation of oxidative phosphorylation by LAA could be a tool to overcome GEM resistance. Clinical verification of the effectiveness of LAA in GEM resistance is necessary in the future.

## 1. Introduction

Pancreatic ductal adenocarcinoma (PDA) is an aggressive malignancy with an extremely poor prognosis. The median survival time is 6 months, and the 5-year survival rate is approximately 9% [1]. In the absence of improved outcomes, PDA is predicted to become the second leading cause of cancer-related deaths within the next decade [2]. Even systemic therapy with gemcitabine (GEM), a deoxycytidine analog, has not increased the median survival of patients beyond 6 months due to the acquisition of chemoresistance [3]. Therefore, there is an urgent need for an effective treatment against this devastating disease as well as clarification of the mechanism underlying chemoresistance to improve therapeutic outcomes.

The hypoxic tumor microenvironment is a research focus as it is a major barrier to successful treatment of PDA. Hypoxia is a hallmark of many types of solid tumors and is associated with disease progression, therapeutic resistance, and poor prognosis [4]. Hypoxic conditions are associated with signaling pathways controlling cell proliferation, angiogenesis, and apoptosis [5], which are linked to resistance to chemotherapy and radiation therapy and, hence, poor prognosis [6]. Intratumoral hypoxia causes reprogramming of energy metabolism, suppression of oxidative phosphorylation (OXPHOS), and promotion of glycolysis and lactate fermentation mediated by hypoxia-inducible factor 1 alpha (HIF-1α), which affects cancer cell properties [7,8]. HIF-1α is also reported to be involved in PDA [9,10].

Stemness and epithelial–mesenchymal transition (EMT) are characteristics of cancer cells under hypoxia [11,12]. It has been reported that there is a subpopulation of cells in PDA that exhibit stem cell-like phenotypes. These cells are termed pancreatic cancer stem cells (CSC) and have the capacity to self-renew and differentiate into heterogeneous cancer cells. CSCs play a key role in tumor initiation, invasion, metastasis, and therapeutic resistance as well as in local recurrence following curative resection [13,14,15]. EMT is characterized by the loss of intercellular adhesion, decreased expression of epithelial markers, including E-cadherin, and enhanced expression of mesenchymal markers, such as vimentin [16]. EMT also plays an important role in tumor invasion, metastasis, and therapeutic resistance. Notably, a direct connection has been reported between EMT and the acquisition of stemness, which suggests a close association between CSC properties and EMT [17]. Moreover, various molecules and signaling pathways that are activated by hypoxia may contribute to the induction of malignant phenotypes of PDA, such as proliferation, invasion, tumorigenesis, chemosensitivity, and autophagy.

Lauric acid (LAA), which is a saturated medium-chain fatty acid with 12 carbon atoms and the primary fatty acid in coconut oil, has been associated with certain health benefits of coconut oil intake. We have previously reported that LAA exerts anticancer effects via oxidative stress [18,19]. However, the effect of LAA on PDA growth remains unclear.

The present study aimed to elucidate the central mechanism of hypoxia-induced chemoresistance in PDA. Furthermore, we evaluated the efficiency of LAA against PDA chemoresistance. The findings of this study are expected to provide a basis for developing therapeutic approaches to target hypoxia-associated factors with an aim to successfully treat refractory PDA.

## 2. Results

### 2.1. Hypoxia-Induced GEM Resistance in MIA-PaCa-2 PDA Cells

**Figure 1:**
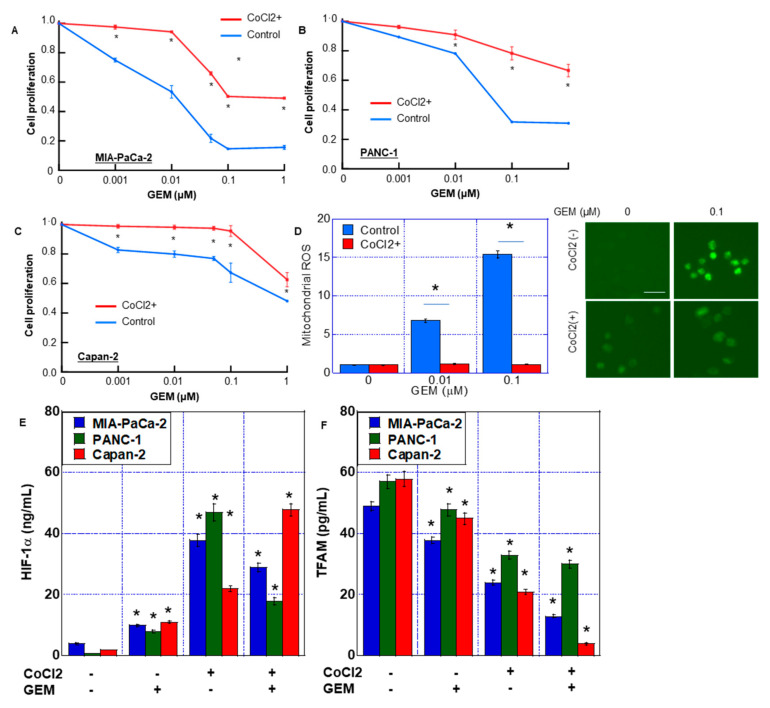
GEM resistance induced by CoCl_2_ in MIA-PaCa-2 PDA cells. (**A**–**C**) Effect of CoCl_2_ on cell proliferation in MIA-PaCa-2 (**A**), Panc-1 (**B**) and Capan-1 (**C**). Cells were treated with CoCl_2_ (150 μM) and GEM for 72 h. (**D**) ROS production was examined by DHR in MIA-PaCa-2. (Right) Fluorescence images of DHR. Scale bar, 50 μm. (**E**,**F**) HIF-1α and TFAM levels in MIA-PaCa-2, Panc-1 and Capan-1. Cells were treated with or without CoCl_2_ (150 μM) and GEM (0.1 μM). Error bars represent the standard deviations from three independent examinations. * *p* < 0.05. (**E**,**F**) vs. CoCl_2_(-)/GEM(-). Statistical differences were calculated by ordinary analysis of variance. GEM, gemcitabine; PDA, pancreatic ductal adenocarcinoma; ROS, reactive oxygen species; DHR, dihydrorhodamine 123; HIF, hypoxia-inducing factor; TFAM, transcription factor A mitochondrial.

First, we examined the effect of HIF-1α on GEM sensitivity (Figure 1). We examined the effect of concurrent treatment with CoCl_2_ and GEM on MIA-PaCa-2 cells (Figure 1A). The sensitivity of GEM was suppressed by CoCl_2_. CoCl_2_ treatment increased the inhibitory concentration IC50 of GEM by 75% from 0.008 to 0.06 μM. Similarly, in the PANC-1 and Capan-2 cell lines, GEM-induced growth inhibition was suppressed by CoCl_2_ (Figure 1B,C). Thus, simultaneous treatment with CoCl_2_ and GEM suppressed the latter’s antitumor effect.

Next, we compared reactive oxygen species (ROS) generation after treatment with GEM, with or without CoCl_2_. ROS derived from mitochondria (DHR and H_2_O_2_) increased in a GEM dose-dependent manner without CoCl_2_. In contrast, CoCl_2_ co-treatment abrogated the induction of mitochondrial ROS by GEM (Figure 1D).

In MIA-PaCa-2 cells, CoCl_2_ and CoCl_2_ + GEM increased HIF-1α protein levels in the nuclear fraction. In addition, CoCl_2_ reduced the TFAM protein level, which was even further reduced by CoCl_2_ + GEM treatment (Figure 1E,F). In PANC-1 and Capan-2 cells, CoCl_2_ and CoCl_2_ + GEM increased the HIF-1α protein levels, whereas CoCl_2_ + GEM decreased the TFAM protein levels.

### 2.2. Hypoxia-Induced Stemness in MIA-PaCa-2 PDA Cells

**Figure 2:**
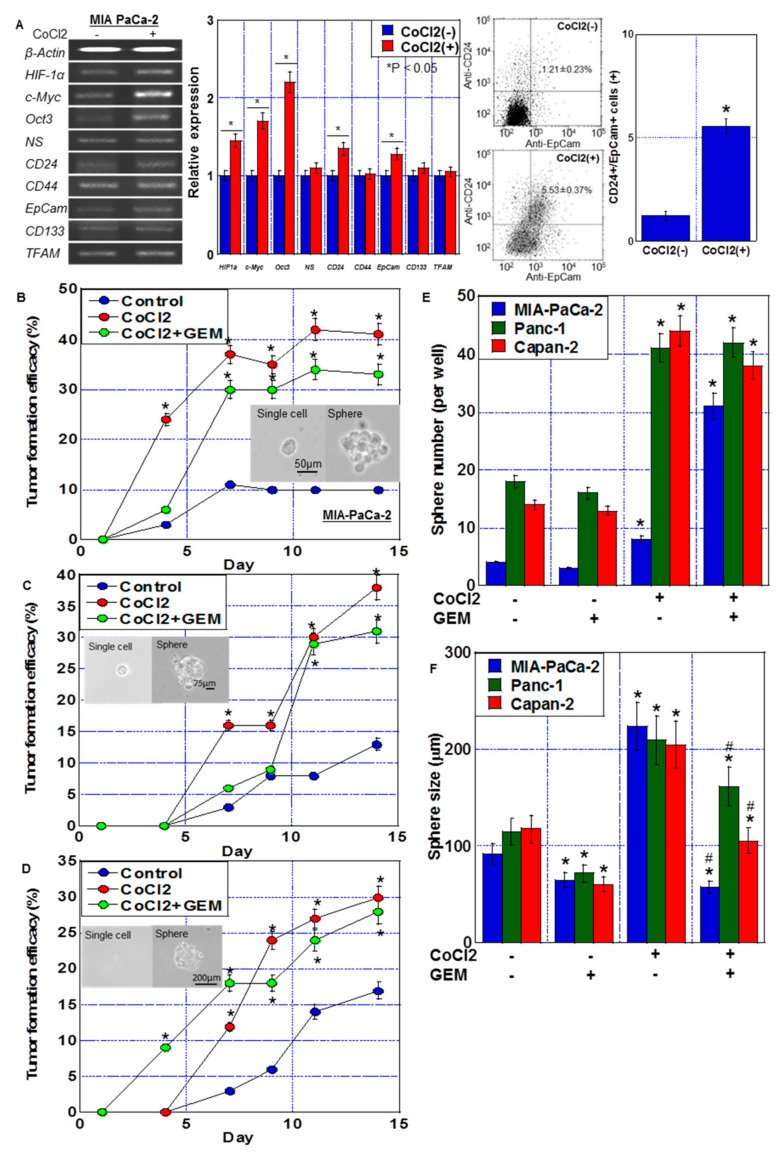
Stemness induced by CoCl_2_ in MIA-PaCa-2 PDA cells. (**A**) Effect of CoCl_2_ (150 μM, 72 h) on mRNA expression of *HIF-1α* and stem cell markers (*c-Myc*, *Oct3*, *NS*, *CD24*, *CD44*, EpCam, *CD133*, and *TFAM*) in MIA-PaCa-2. β-Actin was amplified for loading standard. (Middle) Semi-quantification of mRNA expression. (Right) Flow cytometric analysis of CD24+/EpCam+ cells. (**B**–**D**) Effect of CoCl_2_ and/or GEM on stemness assessed by single-cell sphere formation efficiency in MIA-PaCa-2 (**B**), Panc-1 (**C**) and Capan-1 (**D**). Single adherent cells were seeded in a 96-well plate. Cells were treated with or without CoCl_2_ (150 μM) and GEM (0.1 μM) for 72 h. (Insert) Phase-contrast images of single cell and sphere. Scale bar, 50 μm. (**E**,**F**) Effect of CoCl_2_ and/or GEM on stemness assessed by secondary sphere assay in MIA-PaCa-2, Panc-1 and Capan-1. (**E**) Sphere number. (**F**) Sphere size. Error bars represent the standard deviations from three independent examinations. * *p* < 0.05. (B–D) vs. Control. (**E**,**F**) vs. CoCl_2_(-)/GEM(-). ^#^ *p* < 0.05. (**F**) vs. CoCl_2_. Statistical differences were calculated by ordinary analysis of variance. GEM, gemcitabine; PDA, pancreatic ductal adenocarcinoma; NS, nucleostemin; HIF, hypoxia-inducing factor; EpCam, epithelial cell adhesion molecule; TFAM, transcription factor A mitochondrial.

The mRNA expression of four types of stem cell markers was used to evaluate the effect of CoCl_2_ on stemness (Figure 2A). CoCl_2_ treatment increased the expression of *HIF-1α*, *c-Myc*, *Oct3*, CD24 and EpCam, whereas the expression of *NS*, *CD44*, *CD133*, and *TFAM* was not altered. To examine the stem cell population in MIA-PaCa-2 cells, CD24+/EpCam+ cells were detected by flow cytometry. CoCl_2_ treatment increased CD24+/EpCam+ cell population from 1.21 ± 0.23% to 5.53 ± 0.37%.

The effect of CoCl_2_ on stemness was also examined by single-cell sphere forming efficacy (Figure 2B–D) and secondary sphere formation (Figure 2E,F). Single-cell sphere forming efficacy of PDA cells was enhanced by CoCl_2_ and CoCl_2_ + GEM treatments. CoCl_2_ also enhanced sphere formation in terms of number and size. In contrast, additional GEM treatment reduced the sphere size, but not the CoCl_2_-induced increase in the number of spheres. The number of spheres increased with CoCl2 treatment, while the size decreased with the combined use of GEM. It is conceivable that this was influenced by the suppression of proliferation by GEM. GEM treatment alone also reduced the sphere size.

### 2.3. Energy Metabolism in MIA-PaCa-2 PDA Cells under Hypoxia

**Figure 3:**
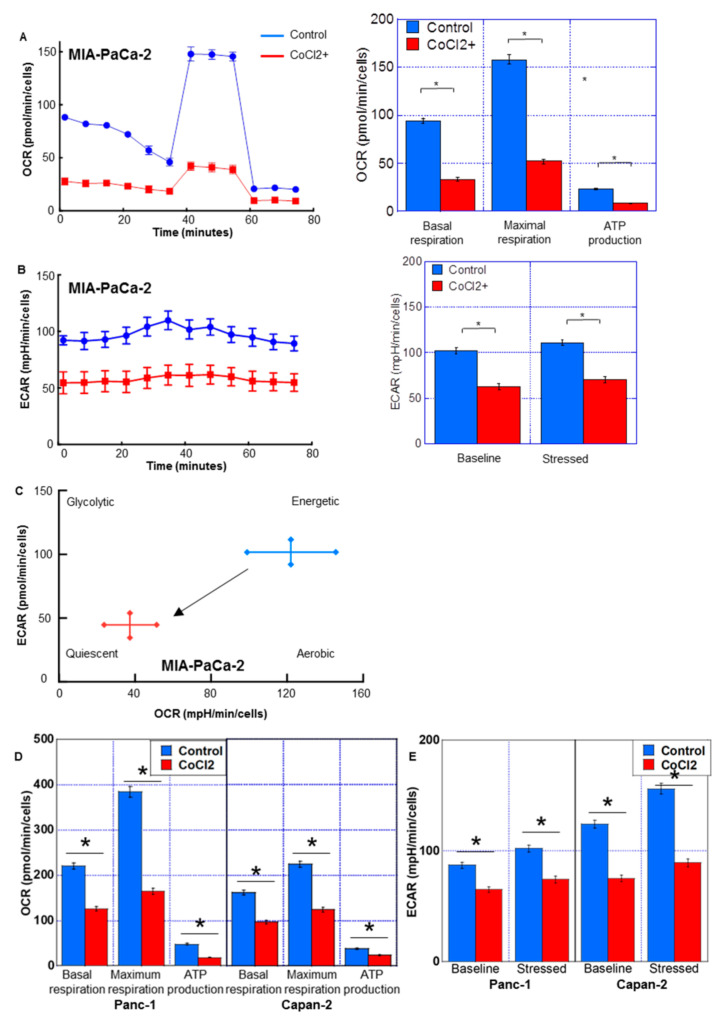
Energy metabolism in MIA-PaCa-2 PDA cells treated with CoCl_2_. Flux analysis of CoCl_2_ (150 μM, 72 h)-treated PDC cells. (**A**,**D**) Effect of CoCl_2_ on the OCR, basal respiration, maximum respiration, and ATP production. (**B**,**E**) The effect of CoCl_2_ on glycolytic activity (ECAR), baseline ECAR, and stressed ECAR (right). (**C**) Effect of CoCl_2_ on the cell energy phenotype profile. Error bars indicate standard error from three trials. * *p* < 0.05. Statistical significance was calculated using ordinary analysis of variance. PDA, pancreatic ductal adenocarcinoma; OCR, oxygen consumption rate; ECAR, extracellular acidification rate.

The effect of CoCl_2_ on the energy metabolism of PDC cells was examined using flux analysis (Figure 3). Basal respiration, maximum respiration, and ATP production were suppressed by CoCl_2_ (Figure 3A,D). Moreover, glycolysis/lactate fermentation, measured by ECAR, was also suppressed by CoCl_2_ in both the baseline and stressed phases (Figure 3B,E). As shown in Figure 3C, the metabolic phenotype profile revealed that CoCl_2_ induced a quiescent phenotype in MIA-PaCa-2 cells.

### 2.4. Establishment of GEM-Resistant PDA Cells by Prolonged Hypoxic Treatment

**Figure 4:**
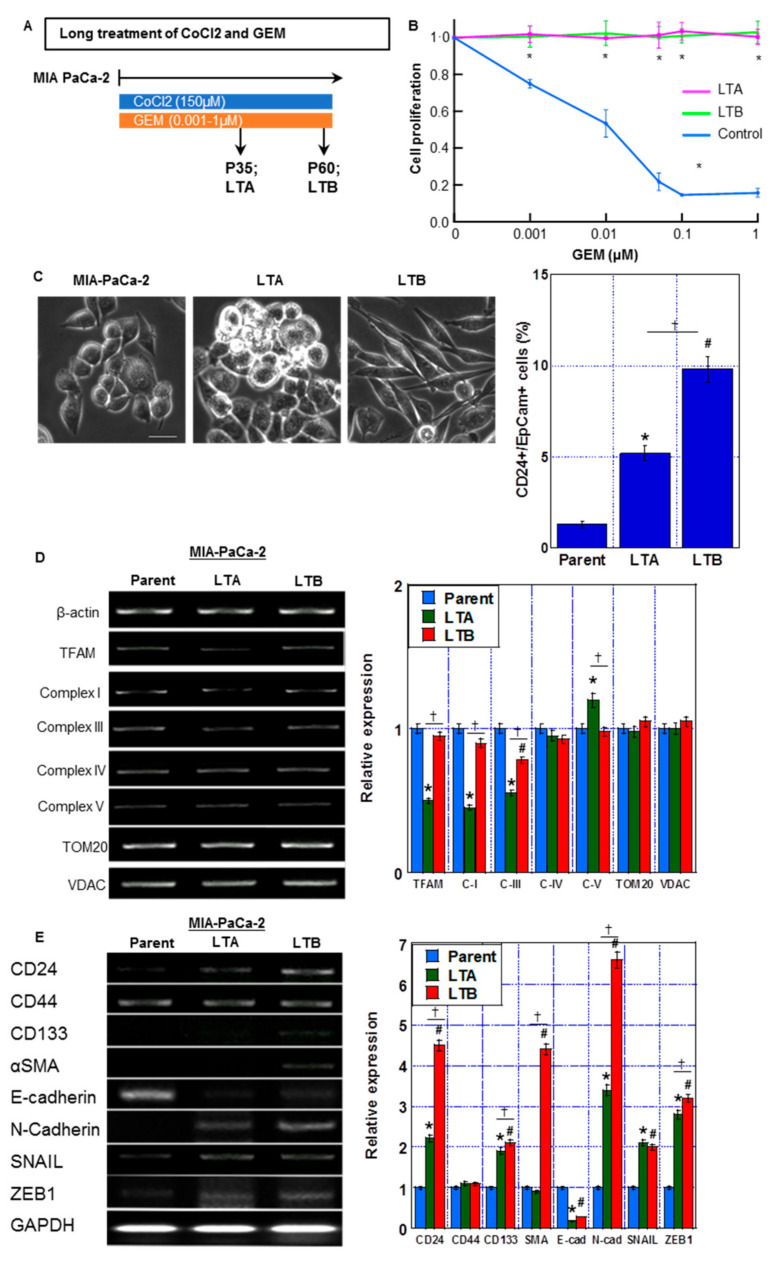
Establishment of GEM-resistant PDA cells by prolonged hypoxic treatment. (**A**) Protocol of establishment of GEM-resistant PDA cells (indicated as long treatment A and B (LTA and LTB) cells) by prolonged treatment of MIA-CaPa-2 parent cells with CoCl_2_ and GEM. (**B**) Cell proliferation in LTA and LTB cells. Cells were treated with GEM for 72 h. (**C**) (Left) The morphological changes under phase-contrast images. Scale bar, 50 μm. (Right) CD24+/EpCam+ cell population by flow cytometric analysis. (**D**) mRNA expression of migochondria-associated genes: TFAM, ND1 (Complex I), Cytb (Complex III), CoI (Complex IV), and ATP6 (Complex V) in LTA and LTB cells were examined by RT-PCR. β-Actin was amplified for loading standard. TOM20 and VDAC were amplified for mitochondrial volume markers. (**E**) mRNA expression of stemness-associated genes: CD24, CD133, α-SMA and SNAIL in LTA and LTB cells were examined by RT-PCR. β-Actin was amplified for loading standard. (**C**–**E**) Cells were cultured without GEM and/or CoCl_2_ treatment. Error bars indicate standard error from three trials. * *p* < 0.05. (**A**) vs. LAA(-). (**C**–**E**) Parent vs. LTA. ^#^ *p* < 0.05. (**C**–**E**) Parent vs. LTB. ^✝^ *p* < 0.05. (**C**–**E**) LTA vs. LTB. Statistical significance was calculated using ordinary analysis of variance. GEM, gemcitabine; PDA, pancreatic ductal adenocarcinoma; LTA, MIA PaCa-2 long treatment A; LTB, MIA PaCa-2 long treatment B.

Next, we established GEM-resistant PDA cells through continuous treatment of MIA-PaCa-2 cells with CoCl_2_ and GEM (Figure 4A). At passage 35 of subculture, long-treated A (LTA) cells were confirmed to have acquired GEM resistance (Figure 4B). Furthermore, at passage 60 of subculture, long-treated B (LTB) cells were confirmed to have acquired GEM resistance (Figure 4B). GEM-induced growth inhibition was completely suppressed at each GEM concentration in both LTA and LTB cells. With regard to their morphologies (Figure 4C), although the MIA-PaCa-2 cells showed a polygonal shape, LTA cells proliferated with marked stratification and LTB cells showed spindle morphology. In LTA and LTB cells, CD24+/EpCam+ cell population was increased.

As shown in Figure 4D, the LTA cells showed a decreased expression of the mitochondria-associated genes *TFAM*, *ND1* (Complex I), and *Cytb* (Complex III), whereas only *Cytb* was decreased in LTB cells in comparison to that in parent MIA-PaCa-2 cells. Furthermore, the expression of stemness-associated genes *CD24*, *CD133*, *SNAIL*, *Zeb1* and *N-cadherin* was increased and the expression of *E-cadherin* was decreased in both LTA and LTB cells in comparison to that in MIA-PaCa-2 cells (Figure 4E). Notably, *CD24* and *CD133* expression was more pronounced in LTB than in LTA cells. Moreover, LTB cells showed higher levels of *α-SMA* than LTA cells.

Thus, the LTA cells showed increased expression of stemness-related genes and a marked decrease in mitochondrial complex genes. In contrast, the LTB cells showed a decrease in expression of only mitochondrial Complex III-related gene and a marked increase in stemness-related gene expression with acquisition of EMT phenotype.

### 2.5. Mitochondrial Impairment by Prolonged Hypoxic Treatment of MIA-PaCa-2 PDA Cells

**Figure 5:**
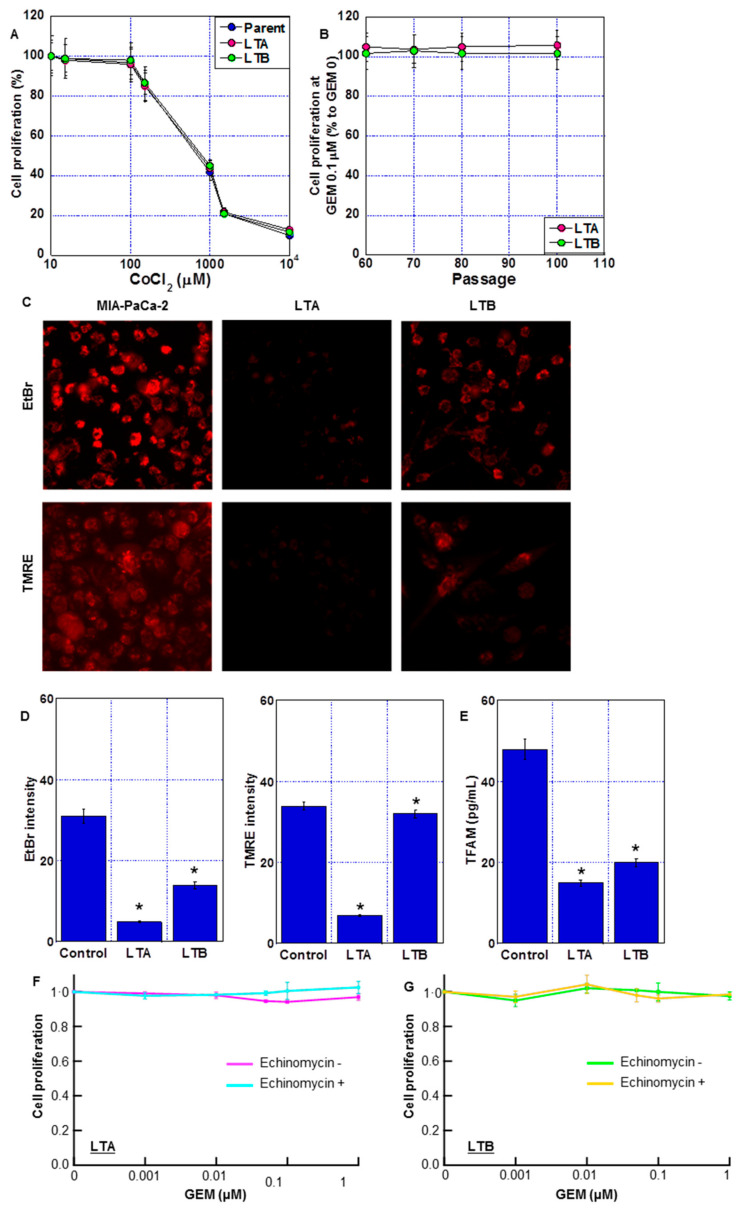
Mitochondrial impairment in long-term hypoxia-induced GEM-resistant PDA cells. (**A**) Effect of CoCl_2_ on cell proliferation of LTA and LTB cells. (**B**) GEM resistance after cessation of GEM and CoCl_2_ treatment. (**C**) Fluorescent images of mitochondria DNA (EtBr) and mitochondrial membrane potential (TMRE). Scale bar, 50 μm. (**D**) Semi-quantification of mitochondrial EtBr and TMRE signals. (**E**) TFAM levels were measured by ELISA. Error bars represent the standard deviations from three independent examinations. (**A**–**E**) Cells were cultured without GEM and/or CoCl_2_ treatment. (**F**,**G**) Effect of echinomycin on GEM sensitivity in LTA and LTB cells. Cells were treated with GEM for 72 h with or without echinomycin (1.2 nM). * *p* < 0.05. (**D**,**E**) vs. Control. Statistical differences were calculated by ordinary analysis of variance. GEM, gemcitabine; PDA, pancreatic ductal adenocarcinoma; LTA, MIA PaCa-2 long treatment A; LTB, MIA PaCa-2 long treatment B; EtBr, ethidium bromide; TMRE, tetrathylrhodamine ethyl ester; TFAM, mitochondrial transcription factor A; ELISA, enzyme-linked immunosorbent assay.

The LTA and LTB cells showed no alteration in CoCl_2_ sensitivity from parent cells (Figure 5A). GEM resistance in both cells was maintained after cessation (passage 60~) of CoCl_2_ and GEM treatment (Figure 5B).

Next, we examined the effect of long-term treatment with CoCl_2_ and GEM on mitochondrial DNA and mitochondrial membrane potential (indicated by TMRE signal). In the EtBr treatment method, only the mitochondrial DNA in the cytoplasm was stained, and the DNA in the nucleus was not stained. The amount of mitochondrial DNA decreased in the LTA and LTB cells (Figure 5C,D), with the LTA cells showing a more prominent decrease. The LTA cells also showed a decreased TMRE signal and TFAM level (Figure 5D,E). In contrast, the TMRE signal was maintained and TFAM levels were only partially reduced in the LTB cells. To eliminate the effect of HIF-1α activation by chemical hypoxia treatment with CoCl_2_, both LTA and LTB cells were treated with echinomycin, an HIF-1α inhibitor (Figure 5F,G); however, GEM sensitivity was not restored.

Thus, LTA cells showed significant mitochondrial impairment compared to ρ0 cells, which are deleted mitochondrial DNA [20]. In contrast, mitochondrial impairment was milder in LTB cells than in LTA cells. This mitochondrial DNA damage may have contributed to the perpetuation of GEM resistance, which persisted even after HIF-1α inactivation.

### 2.6. Energy Metabolism in GEM-Resistant PDA Cells Established by Hypoxia

**Figure 6:**
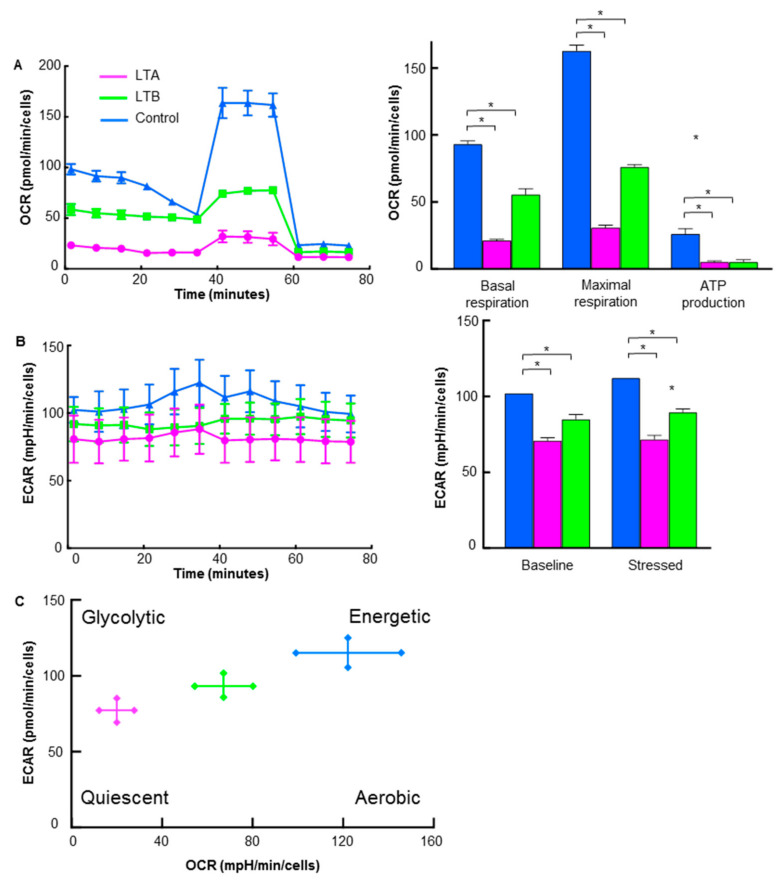
Energy metabolism in GEM-resistant PDA cells. Flux analysis of LTA and LTB cells without CoCl_2_ or GEM treatment. (**A**) OCR (left), basal respiration, maximum respiration, and ATP production (right). (**B**) Glycolytic activity (ECAR; left), baseline ECAR, and stressed ECAR (right). (**C**) Energy phenotypic profiles. (**A**–**C**) Cells were cultured without GEM and/or CoCl_2_ treatment. Error bars indicate standard error from three trials. * *p* < 0.05. Statistical significance was calculated using ordinary analysis of variance. GEM, gemcitabine; PDA, pancreatic ductal adenocarcinoma; LTA, MIA PaCa-2 long treatment A; LTB, MIA PaCa-2 long treatment B; OCR, oxygen consumption rate; ECAR, extracellular acidification rate.

The energy metabolism of LTA and LTB cells was examined using flux analysis (Figure 6). Basal respiration, maximum respiration, and ATP production were all decreased in LTA and LTB cells compared to those in the MIA-PaCa-2 cells (Figure 6A); however, OCR suppression in LTB cells was partial compared with that in LTA cells. ECAR of both baseline and stressed phases was decreased in the LTA and LTB cells in comparison with that in MIA-PaCa-2 cells (Figure 6B); however, ECAR suppression in LTB cells was partial in comparison with that in LTA cells. As shown in Figure 6C, the metabolic phenotype profile showed that LTA cells showed quiescent changes, whereas LTB cells showed fewer quiescent changes.

### 2.7. Effect of LAA on GEM Sensitivity in GEM-Resistant PDA Cells

**Figure 7:**
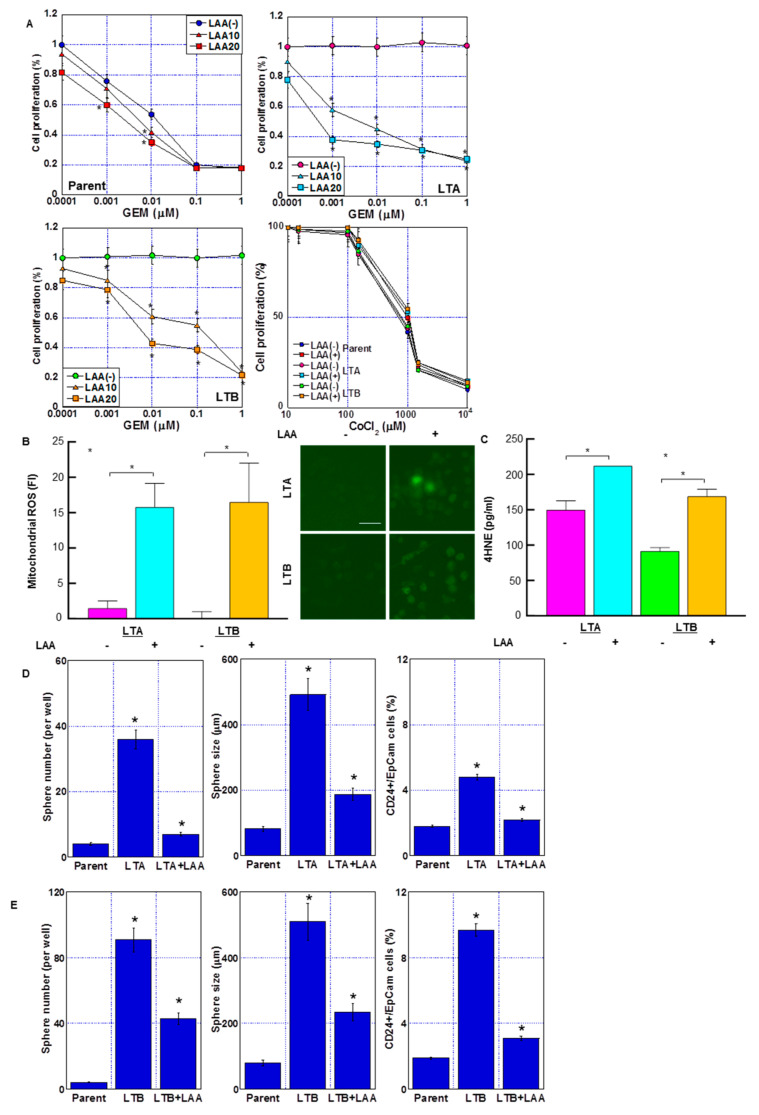
Effect of LAA on GEM sensitivity in GEM-resistant PDA cells. (**A**) Effect of LAA on cell proliferation. Cells were treated with GEM for 72 h with or without LAA (10 and 20 μg/mL). Effect of CoCl_2_ and/or LAA (20 μg/mL) on cell proliferation was also examined. (**B**) Effect of LAA on ROS production. (Right) Fluorescence images of DHR. Scale bar, 50 μm. (**C**) 4-HNE levels measured by ELISA. Error bars indicate standard error from three trials. Statistical significance was calculated using Student’s *t*-test. (**D**,**E**) Stemness of the GEM-resistant PDA cells. Sphere formation in (**D**) LTA and (**E**) LTB cells was assayed, wherein 10,000 cells were treated with or without LAA for 3 days. (Left) Number of spheres. (Middle) Sphere size. (Right) CD24+/EpCam+ cell population by flow cytometric analysis. (**A**–**E**) Cells were cultured without GEM and/or CoCl_2_ treatment. Error bars indicate standard error from three trials. * *p* < 0.05. (**A**) vs. LAA(-), (**D**,**E**) vs. Parent. Statistical significance was calculated using ordinary analysis of variance. GEM, gemcitabine; PDA, pancreatic ductal adenocarcinoma; LTA, MIA PaCa-2 long treatment A; LTB, MIA PaCa-2 long treatment B; LAA, lauric acid; ROS, reactive oxygen species; DHR, dihydrorhodamine 123; FI, fluorescent intensity; ELISA, enzyme-linked immunosorbent assay.

In our previous studies, LAA had a marked antitumor effect on colorectal cancer cells via an increase in mitochondrial ROS production [16,17,18]. We examined the antitumor effect of LAA along with GEM in GEM-resistant LTA and LTB cells (Figure 7). LAA enhanced GEM sensitivity in both LTA and LTB cell lines (Figure 7A). We examined cell growth in the parent, LTA and LTB cells with/without two different concentrations of LAA under various concentrations of GEM. In the results, LAA showed under 20% inhibition of cell growth by 20 μM LAA without GEM, which is not altered among three cell lines. In parental cells, LAA showed an additive effect on GEM, whereas in LTA and LTB cells, LAA showed a synergistic antiproliferative effect with GEM. LTB cells showed residual GEM resistance compared to the LTA cells. Effect of CoCl_2_ on cell proliferation was not affected by LAA.

Next, we examined ROS generation after treatment with GEM and LAA (Figure 7B,C). Mitochondrial ROS (induced by DHR and H_2_O_2_) markedly increased after treatment with GEM and LAA compared to that after GEM alone in LTA and LTB cells (Figure 7B). The 4HNE (a product of lipid peroxidation) concentration also increased after treatment of LTA and LTB cells with GEM and LAA (Figure 7C). LAA reduced the stemness of LTA and LTB cells (Figure 7D,E). Sphere formation was suppressed in both the LAA-treated cell lines in terms of sphere number and size. CD24+/EpCam+ cell population was also decreased by LAA in LTA and LTB cells.

**Figure 8:**
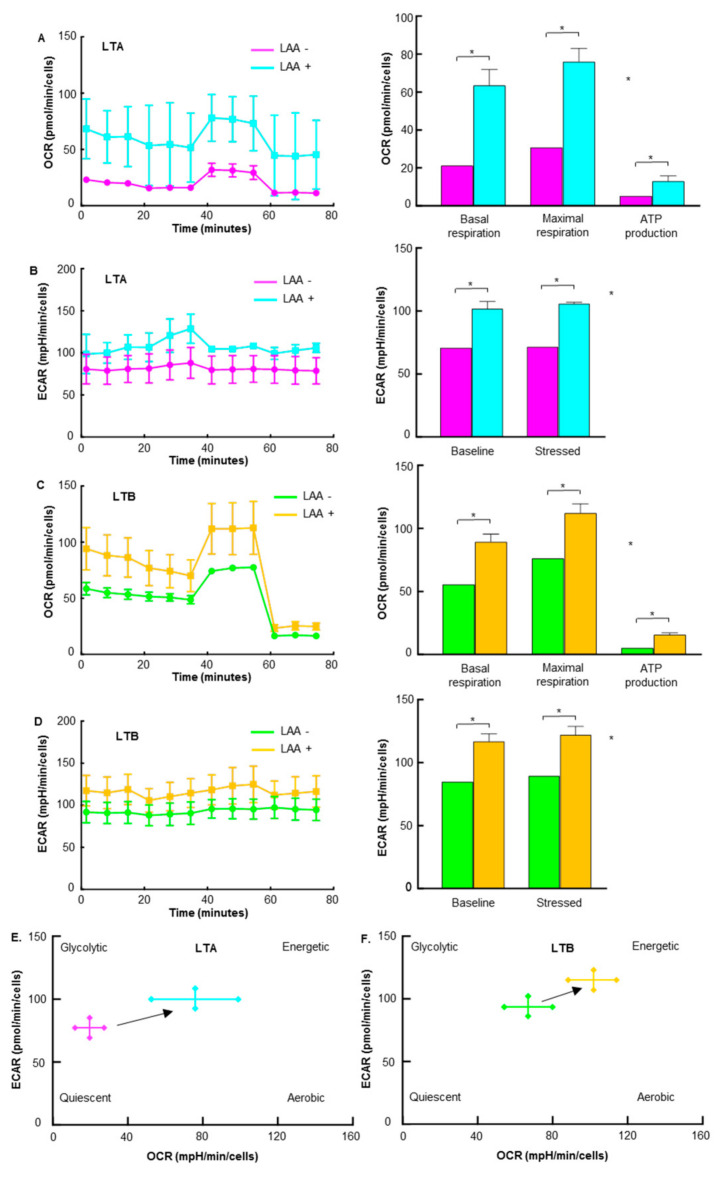
Effect of LAA on energy metabolism in GEM-resistant PDA cells. Flux analysis of LAA (20 μg/mL)-treated LTA and LTB cells. (**A**,**C**) Effect of LAA on the OCR (left), basal respiration, maximum respiration, and ATP production (right) in (**A**) LTA and (**C**) LTB cells. (**C**) Effect of LAA on the energy phenotype profile of LTA cells. (**B**,**D**) The effect of LAA on glycolytic activity (determined by ECAR, left), baseline ECAR, and stressed ECAR (right) in (**B**) LTA and (**D**) LTB cells. (**E**,**F**) Effect of LAA on the energy phenotype profile of (**E**) LTA and (**F**) LTB cells. (**A**–**E**) Cells were cultured without GEM and/or CoCl_2_ treatment. Error bars indicate standard error from three trials. * *p* < 0.05. Statistical significance was calculated using ordinary analysis of variance. GEM, gemcitabine; PDA, pancreatic ductal adenocarcinoma; LTA, MIA PaCa-2 long treatment A; LTB, MIA PaCa-2 long treatment B; LAA, lauric acid; OCR, oxygen consumption rate; ECAR, extracellular acidification rate.

The effect of LAA on energy production in LTA and LTB cells was examined by flux analysis. The OCR, basal respiration, maximum respiration, and ATP production were all increased by LAA in LTA and LTB cells (Figure 8A,C). In LTA, the increase in OCR was more pronounced than that in LTB, and as a result, the levels of OCR in both were similar. The ECAR at baseline and stressed phases was also increased by LAA in LTA and LTB cells (Figure 8B,D). Consequently, the energy profiles of both cells shifted toward energetic from quiescent (Figure 8E,F).

## 3. Discussion

In this study, GEM resistance was induced in pancreatic cancer cell lines by continuous GEM treatment and hypoxia-mimicking CoCl_2_ treatment. In GEM-resistant cell lines, two types of alterations were observed: decreased ROS production due to quiescence energy metabolism and increased stemness and EMT. In contrast, LAA treatment enhanced OXPHOS and promoted ROS production in both GEM-resistant cell lines, resulting in the recovery of GEM sensitivity under both quiescence and stemness/EMT-enhanced conditions.

In this study, hypoxia resulted in GEM resistance in the pancreatic cancer cell lines. A decrease in OXPHOS and suppression of mitochondrial ROS production by GEM were observed. Previous reports have also shown that decreased mitochondrial ROS production is involved in the induction of cisplatin resistance associated with hypoxia [21]. It has also been reported that hypoxia induces antineoplastic resistance by activating the NF-kB pathway and inducing EMT [22]. However, the underlying mechanisms remain unclear. In contrast to another study wherein several GEM-resistant cell lines were established by continuous GEM exposure alone [23], our current study effectively used hypoxia (CoCl_2_) in combination with GEM to establish GEM-resistant cell lines.

The two GEM-resistant cell lines established by continuous CoCl_2_ + GEM treatment showed decreased mitochondrial DNA, TFAM, and expression of mitochondrial-derived genes encoding the electron transport chain complex, and suppression of OXPHOS. These alterations partially resembled those observed in the ρ0 cells. However, unlike true ρ0 cells, stemness was promoted in our established GEM-resistant cells, which carry a partial ρ0 phenotype. It has been previously reported that anticancer drugs cause mitochondrial DNA damage [24]. Our data suggest that the combination of GEM and CoCl_2_ causes strong mitochondrial DNA damage. It has been reported that low oxygen exposure causes damage to both mitochondrial and genomic DNA, and that DNA damage continues in mitochondria with low repair capacity [25]. Mitochondrial DNA mutations also correlate with elevated ROS levels and stabilization of HIF-1α [26,27]. Damage to mitochondrial DNA under hypoxic conditions may involve increased mitochondrial DNA replication capacity associated with elevated levels of oxidation of the D-loop region of mitochondrial DNA under hypoxic conditions [28]. In other words, it is suggested that the replication enhancement associated with hypoxia promotes the damage caused by the antineoplastic agent in the mitochondrial DNA, just as the effect of anticancer drugs appears strongly in the replication-enhanced state, even in the genomic DNA. Thus, antineoplastic agents may cause more severe mitochondrial DNA damage in hypoxic cancer cells such as PDA cells. It is certainly interesting to note that the effect of GEM on CoCl_2_-induced effects on HIF-1α and TFAM varies by cell line. However, in this study, the cause could not be identified. This requires further analysis.

Mutations in the coding region of mitochondrial DNA lead to increased tumorigenicity and metastatic potential [26]. Many cancers carry gene mutations that encode subunits of the electron transport chain [29]. Mutations in the D-loop region of C are associated with hepatocellular carcinoma and prostate cancer, and some neuronal cancers carry mutations in succinate dehydrogenase (Complex II) [30,31].

ROS production was reduced in both GEM-resistant cell lines. Increased ROS production is considered to be part of the cytotoxicity caused by GEM, and decreased ROS production is associated with the promotion of GEM resistance. ROS are cytotoxic owing to their high reactivity [32]; however, at low concentrations, they function as intracellular signal transduction substances that regulate metabolic pathways [33]. The production and release of mitochondrial ROS is a response to cellular stress and acts as a signaling factor that significantly activates HIF, Nrf2, and their downstream gene expression [19].

We showed that CoCl_2_ is involved in GEM resistance by promoting stemness in Panc-1 and Capan-2 as well as in MIA-PaCa-2. CSCs utilize OXPHOS as the preferred energy metabolism and generally exhibit higher oxygen consumption, ROS production, and overall increased mitochondrial function compared to non-stem cells [34]. In addition, mitochondrial ROS promotes KRAS-induced scaffold-independent growth [35]. Conversely, high levels of mitochondrial ROS promote OCT4 degradation and reduce stemness [36]. The results of studies with embryonic stem cells and induced pluripotent stem cells suggest that certain levels of mitochondrial ROS are required to induce cell differentiation, and that low levels of ROS lead to the maintenance of stemness [37]. These results are consistent with previous findings reporting that reduced mitochondrial ROS production correlates with increased stemness [38,39]. In our study, the GEM-resistant cell lines showed reduced ROS levels and enhanced stemness. In mitochondrial damage caused by hypoxia and anticancer drugs, the suppression of OXPHOS is thought to partially reduce mitochondrial ROS production and lead to increased stemness.

In this study, we used CoCl_2_ to mimic hypoxia. Cobalt and nickel inhibit HIF proline hydroxylase and stabilize HIF-1α protein [40]. HIF-1α is a major signaling factor in the biological response to hypoxia [41], and cobalt induces a hypoxic response in cells via HIF-1α activation. Our data also confirmed that cobalt treatment induces HIF-1α production. Hypoxia induces the expression of hypoxic reaction genes and suppresses gene expression via histone hypoacetylation by histone H3 lysine methylation and acetyl-CoA depletion, which are also caused by cobalt treatment [40]. Thus, CoCl_2_ mimics the hypoxic response through a wide range of cellular responses that go beyond the induction of expression of HIF-1α target genes.

HIF-1α acts on BCL2 and p53 to suppress apoptosis [42] and is involved in enhancing stemness in a hypoxic environment [43]. Therefore, whether the results of our study are specific for HIF-1α needs to be examined. The fact that inhibition of HIF-1α in the cell lines established in this study did not restore GEM sensitivity suggests that HIF-1α might not be directly involved in drug resistance in these cell lines. Mitochondrial damage caused by the hypoxic response to HIF-1α may result in HIF1α-independent persistent drug resistance. Although this possibility suggests that HIF-1α targeting might prevent the acquisition of drug resistance due to hypoxia, it might be difficult to abrogate acquired drug resistance by HIF-1α inhibition.

We have previously reported that cancer cells with an imbalance in the electron transport chain complex undergo cell death by promoting OXPHOS and inducing ROS production [16]. LAA treatment has also been associated with EGFR suppression, p53-independent activation of the p21Waf1 apoptosis signal, and promotion of cell death by microRNA [44,45]. Unlike long-chain fatty acids, LAA, which is a medium-chain fatty acid, translocates into the mitochondria in a carnitine shuttle-independent manner and rapidly undergoes β-oxidation [46]. LAA treatment restored GEM sensitivity by suppressing stemness and increasing mitochondrial ROS levels in both quiescent GEM-resistant cells (LTA) and stemness/EMT-enhanced GEM-resistant cells (LTB). After LAA treatment, recovery of higher GEM sensitivity was observed in quiescent LTA cells compared to that in the stemness/EMT-enhanced LTB cells. However, the sphere-forming ability was suppressed by LAA in both cell lines, indicating that LAA inhibits the stemness of cancer cells. LAA-inhibited cancer stemness correlates with suppression of cancer dissemination by LAA in a mouse peritoneal metastasis model [17,18]. LAA is a food nutrient that is abundant in coconut oil [47], and in addition to its antitumor effect, it has been noted for its antibacterial, anti-inflammatory, and cardiovascular improving effects [47,48]. In addition, LAA reduces skeletal muscle atrophy and myocardial damage caused by cancer cachexia [17,18]. PDAs are often complicated with cachexia [49], and therefore LAA is expected to contribute to the reduction in adverse events by chemotherapy in cachectic conditions. In the future, it will be important to investigate the effect on cachexia as well as the rescue effect of GEM susceptibility using animal models. Oral ingestion of LAA as supplemental nutrition during chemotherapy for PDAs can be easily applied clinically, and future studies are awaited.

## 4. Materials and Methods

### 4.1. Cell Line and Reagents

MIA-PaCa-2 human PDA cell line was purchased from Dainihon Pharmaceutical Co. (Tokyo, Japan). PANC-1 and Capan-2 cells were obtained from American Type Culture Collection. Cells were cultured in Dulbecco’s modified Eagle’s medium supplemented with 10% fetal bovine serum at 37 °C in 5% CO_2_. CoCl_2_, echinomycin (WAKO Pharmaceutical, Osaka, Japan) and LAA (Tokyo Chemical Industry, Tokyo, Japan) were purchased.

### 4.2. Cell Growth

Cell growth was assessed using the 3-(4,5-Dimethylthiazol-2-yl)-5-(3-Carboxymethoxyphenyl)-2-(4-Sulfophenyl)-2 H-tetrazolium (MTS) assay as previously described [50]. MTS assays were performed using the CellTiter 96 Aqueous One Solution Cell Proliferation Assay kit (Promega Biosciences, Inc., San Luis Obispo, CA, USA). The plates were read on the Multiskan FC microplate photometer (Thermo Fisher, Tokyo, Japan) at a wavelength of 490 nm. Cells cultured with the control oligonucleotide were used as a control for the MTS assay.

### 4.3. Mitochondrial Imaging

The mitochondrial function was examined using fluorescent probes. Cells were incubated with the probes for 30 min at 37 °C and then imaged using a BZ-X710 all-in-one fluorescence microscope (KEYENCE, Osaka, Japan). We used 10 μM MitoROS (mtROS) (10 μM, AAT Bioquest Inc., Sunnyvale, CA, USA) and 10 μM dihydrorhodamine 123 (DHR; Sigma-Aldrich, St. Louis, MO, USA) to assess oxidative stress, 100 nM MitoGreen (PromoCell GmbH, Heidelberg, Germany) to assess mitochondrial volume, 200 nM tetrathylrhodamine ethyl ester (TMRE; Sigma-Aldrich) to assess mitochondrial membrane potential, and 10 μg/mL ethidium bromide (EtBr; WAKO, for 10 min at room temperature) to assess mitochondrial DNA.

### 4.4. Reverse Transcription Polymerase Chain Reaction (RT-PCR)

Total RNA (1 μg) was used to synthesize cDNA using the ReverTra Ace quantitative PCR (qPCR) RT kit (Toyobo, Osaka, Japan). Then, PCR was performed according to the manufacturer’s instructions. The PCR products were electrophoresed on 2% agarose gels and visualized using EtBr. The primer sets used are listed in Table 1. Primers were synthesized by Sigma-Aldrich (Ishikari, Japan).

### 4.5. Sphere Assay and Tumor Sphere Formation Efficiency (TFE)

Cells (10,000 cells/well) were seeded on uncoated bacteriological 35 mm dishes (Coning Inc., Corning, NY, USA) with 3D Tumorsphere Medium XF (Sigma). After seven days, primary spheres were collected and treated with 2 mM EDTA to dissociate the spheres into individual cells with confirmation that at least 95% of spheres were dissociated. The dissociated cells (500 cells) were seeded in a bacteriological 48-well plate (Coning) with 3D Tumorsphere Medium XF (Sigma) with treatment with CoCl_2_ and/or GEM for 72 h. Sphere images were captured on a computer, and the sphere size was measured. The number of spheres in the culture wells was counted, and the average sphere size from all spheres in the well was determined. To assess single-cell sphere formation efficiency, single adherent cells were seeded in a 96-well plate. The cells were treated with CoCl_2_ or CoCl_2_ + GEM for three days. After incubation, the 3D tumorsphere medium was added to the 96-well plates, and the cells were seeded at 1 cell/well. A typical tumor sphere is >100 μm in diameter and round in shape. The wells containing tumor spheres were counted as positive wells, and those without tumor sphere formation were considered as negative wells.

### 4.6. Enzyme-Linked Immunosorbent Assay (ELISA)

ELISA kits were used to measure the concentration of HIF-1α (Cell Biolabs, Inc., San Diego, CA, USA), TFAM (MyBioSource, Inc., San Diego, CA, USA), and 4-hydroxynonenal (HNE) (Abcam, Cambridge, MA, USA). The assays were performed in accordance with the manufacturers’ instructions.

### 4.7. Mitochondrial Stress Test and Glycolytic Stress Test (Seahorse Assay)

PDC cells were cultured and the oxygen consumption rate (OCR) of 1.5 × 10^4^ viable MIA-PaCa-2 cells per well was measured using a Seahorse XFe24 Extracellular Flux Analyzer with Seahorse XF24 FluxPaks (Agilent Technologies, Santa Clara, CA, USA). Seahorse assays were carried out as follows: OCR in pmol/min was measured before (basal OCR) and after successive injection of 2 μmol/L oligomycin (ATP synthase inhibitor), 1 μmol/L carbonyl cyanide-p-trifluoromethoxy phenylhydrazone (FCCP), an uncoupling protonophore, 0.5 μmol/L rotenone (Complex I inhibitor), and 2.5 μmol/L antimycin A (Complex III inhibitor). From the resulting data, we determined the OCR associated with respiratory ATP synthesis (oligomycin-sensitive) and the maximum OCR in FCCP-uncoupled mitochondria. The extracellular acidification rate (ECAR) was measured using a modified glycolytic stress test in the Seahorse XFe24 Extracellular Flux Analyzer with Seahorse XF24 FluxPaks. Treatment with a combination of rotenone and antimycin allowed the assessment of the impact of electron transport on ECAR by respiratory acidification coupled with the passage of glycolytic pyruvate through the tricarboxylic acid cycle to supply respiration.

### 4.8. Flow Cytometry

To demonstrate cancer stem cells among PDA cells, cell surface markers were analyzed by flow cytometry (FACSCalibur, Becton Dickinson, Franklin Lakes, NJ, USA). Single-cell suspensions of PDA cells in phosphate-buffered saline (PBS) were exposed to antibodies directly coupled with a fluorochrome for 30 min on ice. The antibodies used were anti-human CD24 (Becton Dickinson) coupled to phycoerythrin and anti-human EpCam (Becton Dickinson) coupled to fluorescein-5-isothiocyanate.

### 4.9. Statistical Analysis

Statistical significance was calculated using ordinary ANOVA with the InStat software (version 8.0; GraphPad Software, Inc., San Diego, CA, USA). Data are expressed as the median with 95% confidence interval of three independent experiments. Statistical significance was set at *p* < 0.05 (two-sided).

## Figures and Tables

**Table 1:** Primary sets.

Gene	Accession No.		Sequence
*β-Actin*	NM_001101.3	Upper	GGACTTCGAGCAAGAGATGG
		Lower	AGCACTGTGTTGGCGTACAG
*HIF-1α*	AF208487.1	Upper	GAAAGCGCAAGTCCTCAAAG
		Lower	TGGGTAGGAGATGGAGATGC
*c-Myc*	NM_002467.4	Upper	TTCGGGTAGTGGAAAACCAG
		Lower	CAGCAGCTCGAATTTCTTCC
*Oct3*	BC117437.1	Upper	GAAGGATGTGGTCCGAGTGT
		Lower	GTGAAGTGAGGGCTCCCATA
*NS*	BC001024.2	Upper	ATTGCCAACAGTGGTGTTCA
		Lower	AATGGCTTTGCTGCAAGTTT
*CD24*	BC064619.1	Upper	ATGGGCAGAGCAATGGTG
		Lower	ACCACGAAGAGACTGGCTGT
*CD44*	FJ216964.1	Upper	AAGGTGGAGCAAACACAACC
		Lower	AGCTTTTTCTTCTGCCCACA
*EpCam*	NM_002354.3	Upper	GCTGGTGTGTGAACACTGCT
		Lower	ACGCGTTGTGATCTCCTTCT
*CD133*	BC012089.1	Upper	TTGTGGCAAATCACCAGGTA
		Lower	TCAGATCTGTGAACGCCTTG
*TFAM*	EU279428.1	Upper	CCCCCACAAACCCCATTACTAAACCCA
		Lower	TTTCATCATGCGGAGATGTTGGATGG
*Complex I (ND1)*	YP_003024026.1	Upper	ATGGCCAACCTCCTACTCCT
		Lower	GCGGTGATGTAGAGGGTGAT
*Complex III (Cytb)*	YP_003024038.1	Upper	TATCCGCCATCCCATACATT
		Lower	GGTGATTCCTAGGGGGTTGT
*Complex IV (CO I)*	YP_003024028.1	Upper	ACGTTGTAGCCCACTTCCAC
		Lower	CATCGGGGTAGTCCGAGTAA
*Complex V (ATP6)*	YP_003024031.1	Upper	TATTGATCCCCACCTCCAAA
		Lower	GATGGCCATGGCTAGGTTTA
*TOM20*	NM_014765.3	Upper	ATGGTGGGTCGGAACAGC
		Lower	TCTTCAGCCAAGCTCTGAGC
*VDAC*	L06328.1	Upper	CAGGTACCAACTGCACTCGT
		Lower	CCTTGTGGCCTCCAGCATTA
*αSMA*	BC093052.1	Upper	ACTGCCTTGGTGTGTGACAA
		Lower	TCCCAGTTGGTGATGATGCC
*SNAIL*	NM_005985.3	Upper	ACCCCACATCCTTCTCACTG
		Lower	TACAAAAACCCACGCAGACA
*ZEB1*	NM_001128128.3	Upper	TGCACTGAGTGTGGAAAAGC
		Lower	TGGTGATGCTGAAAGAGACG
*E-cadherin*	Z13009.1	Upper	TGCCCAGAAAATGAAAAAGG
		Lower	GTGTATGTGGCAATGCGTTC
*N-cadherin*	X57548.1	Upper	GACAATGCCCCTCAAGTGTT
		Lower	CCATTAAGCCGAGTGATGGT
*GAPDH*	BC025925.1	Upper	GAGTCAACGGATTTGGTCGT
		Lower	TTGATTTTGGAGGGATCTCG

## Data Availability

Not applicable.

## Abbreviations

GEM, gemcitabine; PDA, pancreatic ductal adenocarcinoma; LAA, lauric acid; OXPHOS, oxidative phosphorylation; HIF-1α, hypoxia-inducible factor 1 alpha; EMT, epithelial-mesenchymal transition; CSC, cancer stem cells; ROS, reactive oxygen species.
